# The teleost inflammasome as a biosensor in developmental toxicology

**DOI:** 10.3389/ftox.2026.1952628

**Published:** 2026-10-19

**Authors:** Darlan Gusso, Manuela Jacobina Sa, Larissa Alessandra Bafoni, Monica Lopes-Ferreira, Carla Lima

**Affiliations:** Plataforma Zebrafish from the Laboratory of Applied Toxinology (CeTICs/FAPESP), Butantan Institute, SãoPaulo, Brazil

**Keywords:** biomarkers, developmental toxicity, ecotoxicology, gasdermin cleavage, IL-1β, inflammasome, xenobiotics, zebrafish embryo assay

## Abstract

**Introduction:**

Standard environmental risk assessment frameworks, such as the OECD 236 Fish Embryo Acute Toxicity (FET) test, rely heavily on macroscopic morphological endpoints that may fail to detect subtle, sublethal molecular disruptions before phenotypic damage becomes apparent. Mechanism-based biomarkers capable of ultra-early toxicity prediction are therefore needed. The teleost inflammasome, particularly the interleukin-1 beta (IL-1β) and gasdermin (GSDM) execution pathway, represents a promising high-resolution molecular biosensor for developmental toxicity screening.

**Methods:**

A systematic review was conducted to evaluate evidence on the teleost inflammasome and the IL-1β/GSDM pathway in response to biological and abiotic stressors, with particular emphasis on their potential application as mechanism-based biomarkers of developmental toxicity.

**Results:**

Available evidence indicates that IL-1β signaling, traditionally characterized as an antimicrobial defense mechanism, can also respond to cellular stress induced by abiotic stressors, including heavy metals, pesticides, and persistent organic pollutants. Sublethal xenobiotic exposure can induce oxidative stress and reactive oxygen species overproduction, leading to the release of damage-associated molecular patterns (DAMPs) and activation of the canonical NLRP3/ASC/caspase-1 platform. Following teleost-specific whole-genome duplication, gasdermin paralogs expanded into distinct variants, including GSDMEa, GSDMEb, and GSDMEc. N-terminal proteolytic cleavage of GSDMEa and GSDMEb generates GSDMEa-N and GSDMEb-N fragments that can mediate pyroptotic pore formation and IL-1β release. However, existing ecotoxicological studies rely predominantly on transcriptional (mRNA) data and rarely verify functional gasdermin protein cleavage.

**Discussion:**

The available evidence supports the IL-1β/GSDM axis as a potentially sensitive molecular indicator of cellular stress and developmental toxicity in teleosts. Transitioning from descriptive morphological assessment toward functional proteomic profiling of GSDMEa/b cleavage, particularly in *Danio rerio* embryos, may provide an additional mechanistic window for complementing emerging mechanism-based approaches and potentially improving the sensitivity and resolution of developmental toxicity assessment.

## Graphical Abstract

**Figure:**
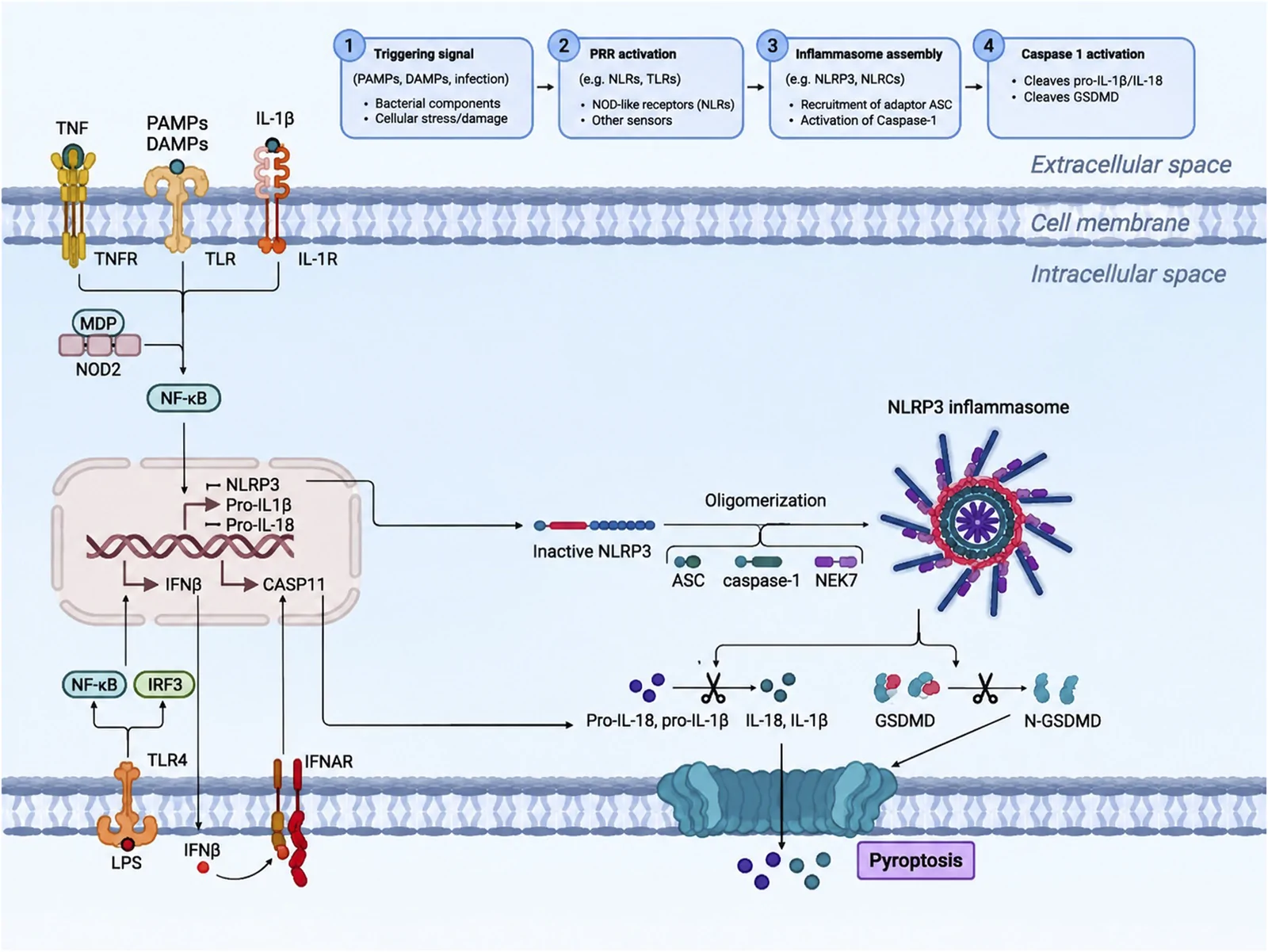
Schematic overview of the NLRP3 inflammasome activation pathway and downstream signaling cascades. The diagram illustrates the priming signal mediated by membrane receptors (such as TLRs and TNFR) leading to NF-_k_b activation and gene transcription, followed by the activation signal triggering the assembly of the NLRP3 inflammasome complex. Subsequent caspase-1 activation results in the cleavage of pro-cytokines (pro-IL-1β and pro-IL-18) and Gasdermin D (GSDMD). The formation of N-GSDMD pores in the plasma membrane mediates cytokine release and the execution of pyroptosis. Note: This is an illustrative diagram; inflammasome signaling pathways may exhibit variations across the more than 50 fish species analyzed in this study.

## 1 Introduction

The accelerating influx of anthropogenic contaminants into aquatic ecosystems represents a major threat to global biodiversity and aquatic animal health. Among these stressors, chemical xenobiotics—particularly pesticides and heavy metals—pose a disproportionate risk to teleost fish during their highly vulnerable early life stages (Meador and Escher, 2025; Sefali et al., 2026). Consequently, developmental toxicity screenings have become indispensable components of environmental risk assessment. Standard regulatory frameworks, such as the Fish Fish Embryo Acute Toxicity (FET) test (OECD, 2013), rely heavily on macroscopic endpoints, including mortality, hatching success, and structural malformations. While effective for identifying gross toxicological defects, these morphological criteria often fail to capture subtle, sublethal molecular disruptions that occur prior to phenotypic damage. There is, therefore, an urgent need to transition from descriptive morphology toward mechanism-based, high-resolution biomarkers capable of providing ultra-early and highly predictive outputs.

A promising yet historically underutilized candidate for high-sensitivity molecular profiling is the teleost innate immune machinery, specifically the interleukin-1 beta (IL-1β) maturation pathway. Canonically, IL-1β activation is mediated by inflammasomes—multiprotein cytosolic complexes comprising an inflammasome sensor, such as NLRP3, NLRC3, or NLRC4 in some teleosts, the adaptor protein ASC (apoptosis-associated speck-like protein containing a CARD), and pro-caspase-1. Furthermore, gasdermin (GSDMD) serves as a key executioner in downstream inflammasome signaling and pyroptotic cell death (Li et al., 2018; Li H. et al., 2022; Li X. et al., 2022 ; Chen S. et al., 2021; Moraleda et al., 2021; Morimoto et al., 2021; Chang, 2023; Mahapatra et al., 2023; Wang H. et al., 2023; Dinakaran et al., 2024; Lu et al., 2024; Ou et al., 2024; Zhao et al., 2024; Hu et al., 2025; Liu et al., 2025a; 2025b; Yañez et al., 2025; Mathiessen et al., 2026). Although traditionally characterized as an evolutionary defense against microbial infection, the teleost inflammasome has recently emerged as an extraordinarily plastic molecular biosensor (Wang D. et al., 2021; Nie et al., 2025; Yang et al., 2025). Acting as an intracellular danger sensor, it undergoes robust cross-activation in response to chemical, physical, and cellular stressors, placing it at the critical intersection of immunomodulation and environmental toxicology.

Research on teleost innate immunity and inflammasome mechanisms has expanded substantially in recent years, with *Danio rerio* representing one of the most extensively studied models alongside a range of economically important aquaculture species. The prominence of *D. rerio* provides a useful experimental framework for investigating these inflammatory pathways, given its fully annotated genome, optical transparency during embryogenesis, and suitability for genetic manipulation and high-throughput assays. These characteristics make zebrafish embryos a valuable model for characterizing inflammatory and cellular stress responses and for exploring their potential relevance to developmental toxicity screening.

Despite this recent research momentum, two critical gaps remain in the integration of the teleost inflammasome into toxicological assessment. First, although IL-1β is widely used as a marker of inflammation, there is a lack of systematic synthesis evaluating how this pathway responds to abiotic stressors, including pesticide and heavy metal exposure. Second, the current literature relies predominantly on transcriptional data (mRNA expression), highlighting the need for functional validation at the protein level, particularly through assessment of downstream Gasdermin cleavage. Following the teleost-specific Whole Genome Duplication (WGD) event (Parey et al., 2022), gasdermin (GSDM) paralogs expanded into distinct variants, including GSDMEa, GSDMEb, and GSDMEc, which may exhibit differential regulation and functional responses in inflammatory and pyroptotic pathways (Jiang et al., 2019; Zhao et al., 2022). This molecular diversification further highlights the importance of moving beyond transcript-level observations toward direct assessment of protein processing and functional activation. Therefore, future studies incorporating protein-level analysis of Gasdermin and caspase cleavage may help clarify how transcriptional responses translate into functional inflammatory and pyroptotic outcomes.

To characterize these knowledge gaps, this study provides a comprehensive systematic and bibliometric review of the literature published between 2020 and 2026, mapping the structural and functional conservation of the teleost inflammasome under both biotic and abiotic challenges. By synthesizing the activation kinetics of the IL-1β/Gasdermin axis, we highlight the potential of profiling the proteolytic cleavage of GSDMEa/b in *D. rerio* embryos to provide an underexplored mechanistic window. Ultimately, integrating mechanism-based inflammasome profiling with conventional descriptive morphology may complement broader next-generation approaches to developmental toxicity assessment by providing additional mechanistic resolution, although its predictive value requires further experimental validation.

## 2 Methods

### 2.1 Literature search and study selection

The literature search was conducted across the Web of Science (3,623 articles) and PubMed (3,357 articles) databases using a predefined set of keywords (detailed below in Supplementary Table S1 – worksheet 1). These keywords were designed to target the innate immune response in teleost fish, with a specific focus on components of the inflammasome complex, cytokines, and pattern recognition receptors. The initial search yielded a total of 6,980 articles published from 2020 to 10 March 2026. Data organization and curation were performed by three independent reviewers using Rayyan software. This rigorous screening process included the removal of duplicate records (n = 2,524) and an exploratory analysis to exclude studies published before 2020, as well as those that did not focus on fish, inflammasomes, or innate immunity (n = 3,654). Following this procedure, 801 eligible articles remained (Supplementary Table S1 – worksheet 2).

### 2.2 Data extraction and synthesis

Following the initial screening, 346 articles underwent full-text assessment to confirm eligibility. An additional manual review was then conducted independently by three reviewers to determine whether the target molecules had been experimentally evaluated in response to a biotic or abiotic stimulus. Articles were excluded if they did not investigate any of the target molecules or only mentioned them without experimental evaluation. This process resulted in the exclusion of 76 additional articles, yielding a final selection of 270 articles for data extraction and qualitative synthesis (Supplementary Table S2). The target molecules included Toll-like receptors (TLRs), myeloid differentiation primary response 88 (MYD88), tumor necrosis factor (TNF), interferons (IFNs), nucleotide-binding oligomerization domain (NOD), interleukin-1 receptor-associated kinase (IRAK), TNF receptor-associated factor (TRAF), ASC, NLRCs, NLRPs, caspases, interleukins, gasdermins, and CXCL. Consensus software was used as an auxiliary tool to facilitate literature screening and data extraction. The extracted variables included species (scientific and common names), stimulus dose/concentration, administration route, analyzed tissue or cell line, target molecule (immune marker), expression level (mRNA or protein), time post-stimulation/infection, direction of regulation, and citation. The three reviewers independently assessed study eligibility using these predefined criteria, and no discrepancies occurred during the screening process because the eligibility assessment was based on objective criteria, particularly the presence or absence of experimental evaluation of the target molecules in response to biotic or abiotic stimuli.

### 2.3 Data sources and analytical procedures for each figure

Figure 1 was constructed based on a systematic literature search in the PubMed and Web of Science databases to identify relevant studies investigating the innate immune system and inflammasomes in fish models. Data were processed and visualized using R software (version 4.4.0). Figure 2 was generated from the dataset provided in Supplementary Table S4, which comprises the curated list of all included studies and their respective metadata. Data visualization and processing were conducted in R software (version 4.4.0). A heatmap of log1p-transformed annotation counts (ranging from 0 to >3, corresponding to raw literature counts of 0–∼54) was plotted. The predominant light blue color across the heatmap highlights a global pattern of data scarcity in the literature. Figure 3 was derived from Supplementary Table S6, utilizing the “cyprinid pathogens” and “non-cyprinid pathogens” spreadsheets. To map the regulatory landscape of interleukin-1 beta (IL-1β) across different teleost species, the extracted data were categorized and visualized as bubble charts using the *ggplot2* package in R software (version 4.4.0). Datasets were stratified by phylogenetic and physiological criteria into two main groups: cyprinids and non-cyprinid teleosts. For each plot, the x-axis denotes the specific stimulus (e.g., bacteria, viruses, parasites, or pathogen-associated molecular patterns [PAMPs] such as peptidoglycan [PGN] and LP), while the y-axis represents the evaluated teleost species. The qualitative IL-1β expression response was color-coded into three distinct categories: upregulated/increased (green), downregulated/decreased (red), or mixed/variable (blue). The biological level of analysis was indicated by symbol fill: mRNA expression (filled circles) *versus* protein quantification (empty circles). Bubble size (count) correlates directly with the absolute number of independent studies reporting that specific host-stimulus interaction, ranging from 1 to ≥5.

**FIGURE 1:**
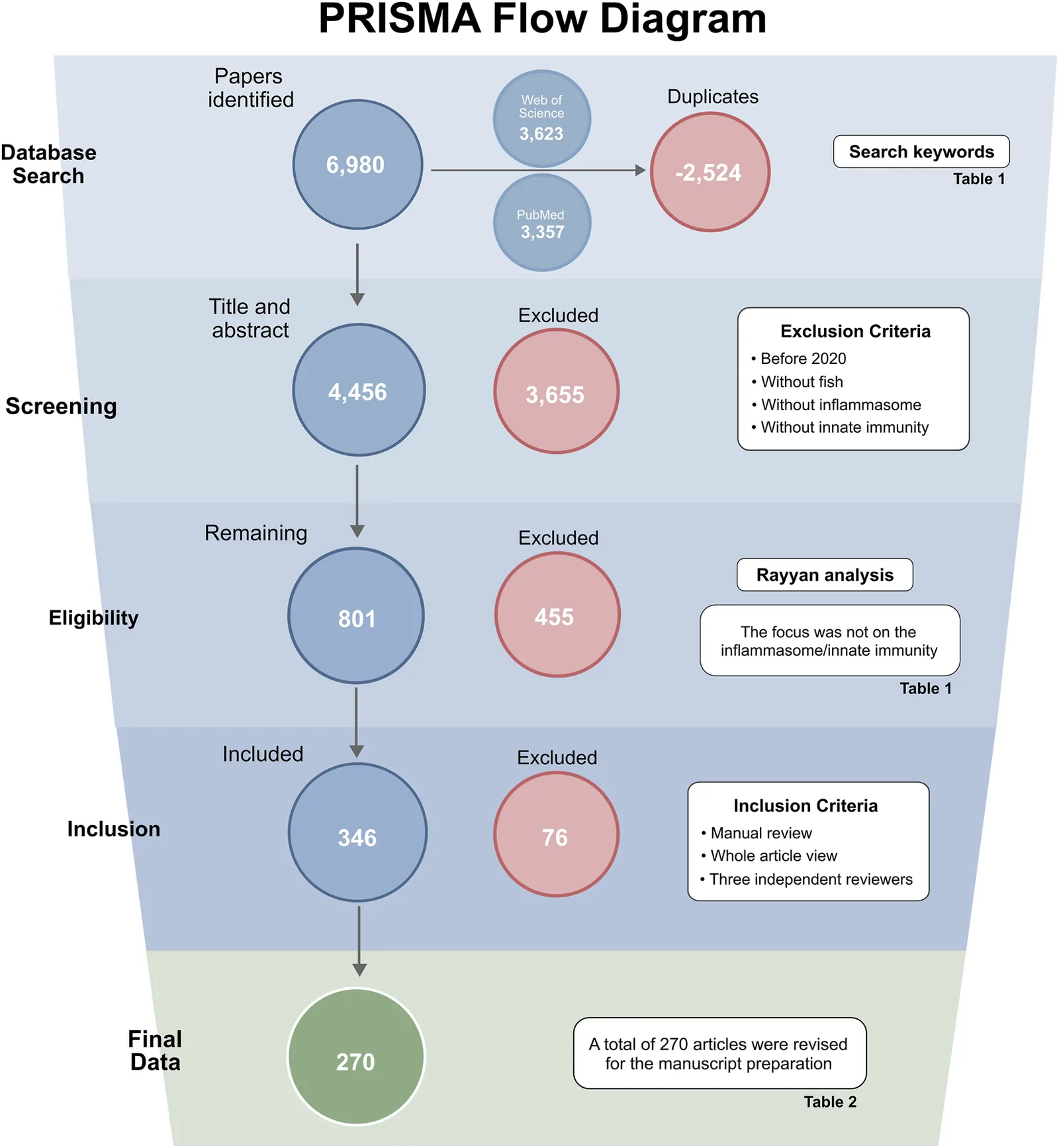
Flowchart of the systematic literature review process. Sequential steps of database searching, screening, eligibility assessment, and final study inclusion. Initial searches across Web of Science (n = 3,623) and PubMed (n = 3,357) yielded 6,980 records. Following duplicate removal (n = 2,524), 4,456 titles and abstracts were screened, resulting in the exclusion of 3,654 records based on predefined criteria (absence of focus on fish, inflammasomes, or innate immunity). The remaining 801 articles underwent eligibility assessment using Rayyan software, leading to 455 exclusions and 346 retained articles. Full-text evaluation by three independent reviewers excluded an additional 76 articles, leaving a final targeted subset of 270 articles for manuscript synthesis.

**FIGURE 2:**
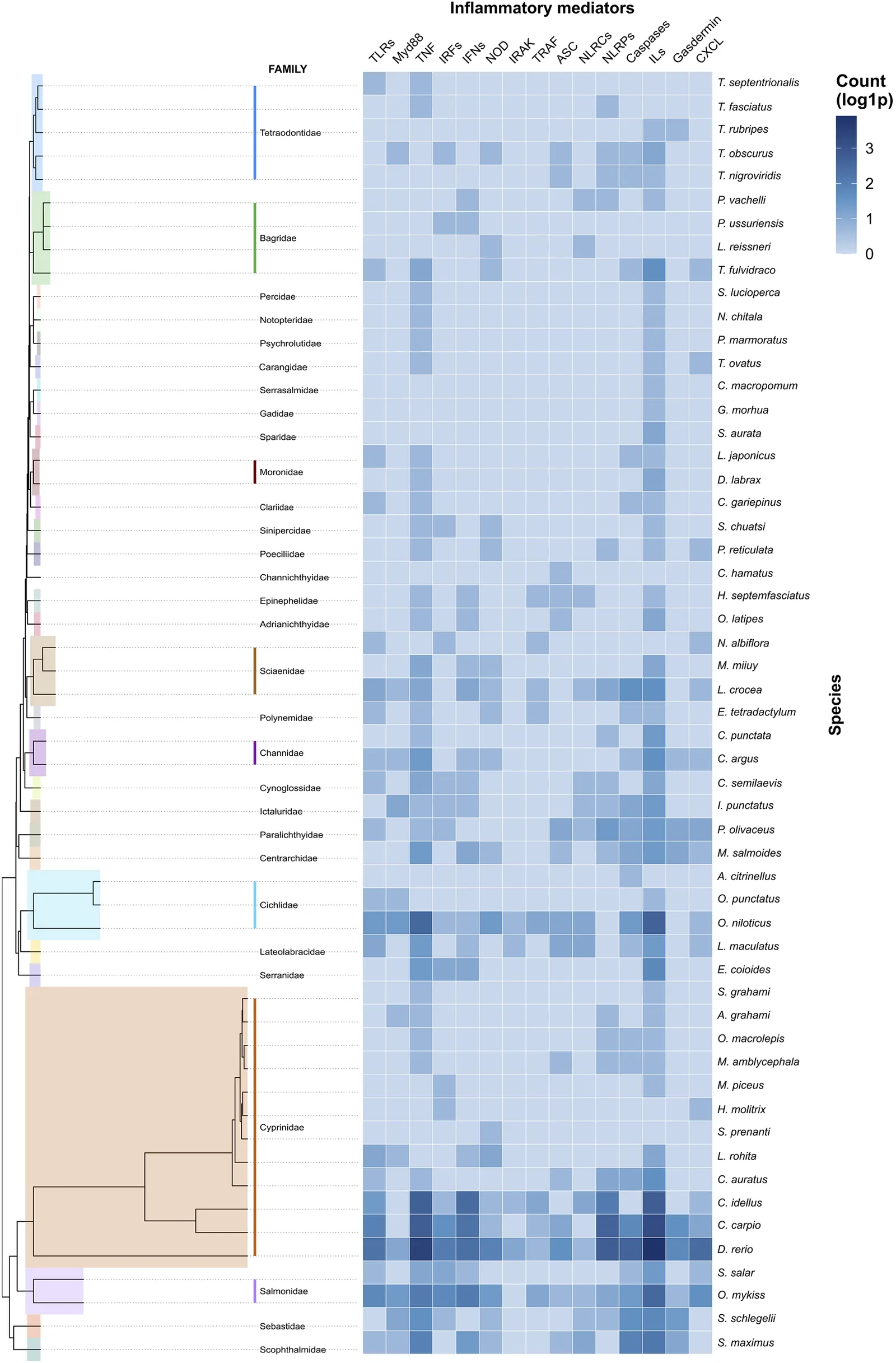
Distribution and publication frequency of innate immune and inflammatory markers across fish species. Heatmap displaying the publication volume/data points for key immune components (TLRs, MyD88, TNF, IRFs, IFNs, NOD, IRAK, TRAF, ASC, NLRCs, NLRPs, Caspases, ILs, Gasdermin, and CXCL) across teleost models, hierarchically grouped by family profile similarity. The extensively characterized Cyprinidae cluster (shaded lower bracket) is highlighted against non-cyprinid lineages. Color gradient (log1p scale: light blue = low/zero counts; dark blue = high counts) denotes literature volume, demonstrating data concentration in model and commercial species (*Danio rerio*, *Ctenopharyngodon idella*, and *Cyprinus carpio*), particularly within TLR, Caspase, and IL pathways.

**FIGURE 3:**
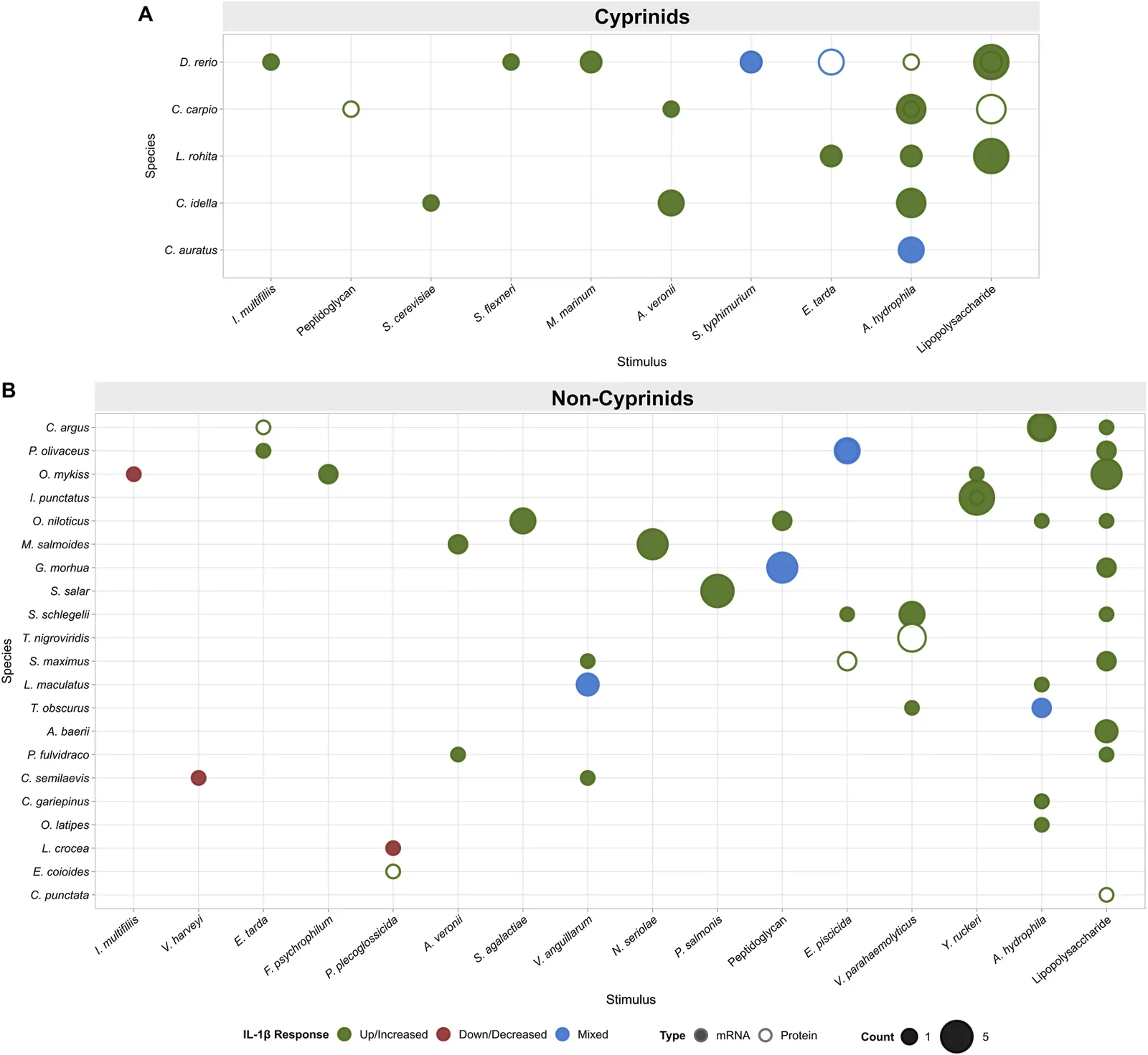
Characterization of the IL-1β response to pathogen-associated molecular patterns (PAMPs) and infectious stimuli in teleosts. **(A)** Bubble plot summarizing IL-1β regulation, expression level, and study volume across five key cyprinid species (*Danio rerio*, *Cyprinus carpio*, *Labeo rohita*, *Ctenopharyngodon idella*, and *Carassius auratus*) exposed to pathogens, cellular components, and inflammatory triggers (e.g., *Ichthyophthirius multifiliis*, peptidoglycan, *Saccharomyces cerevisiae*, *Shigella flexneri*, *Mycobacterium marinum*, *Aeromonas veronii*, *Salmonella serovar typhimurium*, *Edwardsiella tarda*, *Aeromonas hydrophila*, and lipopolysaccharide [LPS]). **(B)** Bubble plot mapping IL-1β dynamics across diverse cyprinid and non-cyprinid teleosts (*Oncorhynchus mykiss*, *Oreochromis niloticus*, *Salmo salar*, *Ictalurus punctatus*, and *Paralichthys olivaceus*) challenged with broad pathogen and immunostimulant panels (e.g., *I. multifiliis*, *Vibrio harveyi*, *E. tarda*, *Flavobacterium psychrophilum*, *Pseudomonas plecoglossicida*, *A. veronii*, *Streptococcus agalactiae*, *Vibrio anguillarum*, *Nocardia seriolae*, *Piscirickettsia salmonis*, peptidoglycan, *Edwardsiella piscicida*, *Vibrio parahaemolyticus*, *Yersinia ruckeri*, LPS, and *A. hydrophila*). Circle color indicates directional response (green: upregulation/increase; red: downregulation/decrease; blue: mixed). Symbol fill distinguishes detection level (solid: mRNA; hollow: protein). Bubble size reflects cumulative independent study count (1 to ≥5).

Figure 4 was generated using Supplementary Table S7 to analyze the functional architecture of teleost inflammasomes. A targeted screening identified studies employing experimental interventions linked to pathogenic or metabolic challenges. IL-1β modulation data were compiled into a comprehensive bubble chart using the *ggplot2* package in R software (version 4.4.0). The y-axis categorizes experimental interventions, encompassing genetic modifications (e.g., knockout, siRNA/dsRNA knockdown, plasmid-mediated overexpression), nutritional shifts (e.g., high-fat diets), and pharmacological or biochemical treatments (e.g., melatonin, silibinin, vitamin D3). The x-axis indicates the tested teleost species. The qualitative dynamics of IL-1β expression were color-coded into upregulated (green), downregulated (red), or mixed responses (blue). Transcriptional (mRNA) and translational/secretory (protein) levels were distinguished by filled and empty circles, respectively, while bubble size denotes the number of independent experimental observations per condition.

**FIGURE 4:**
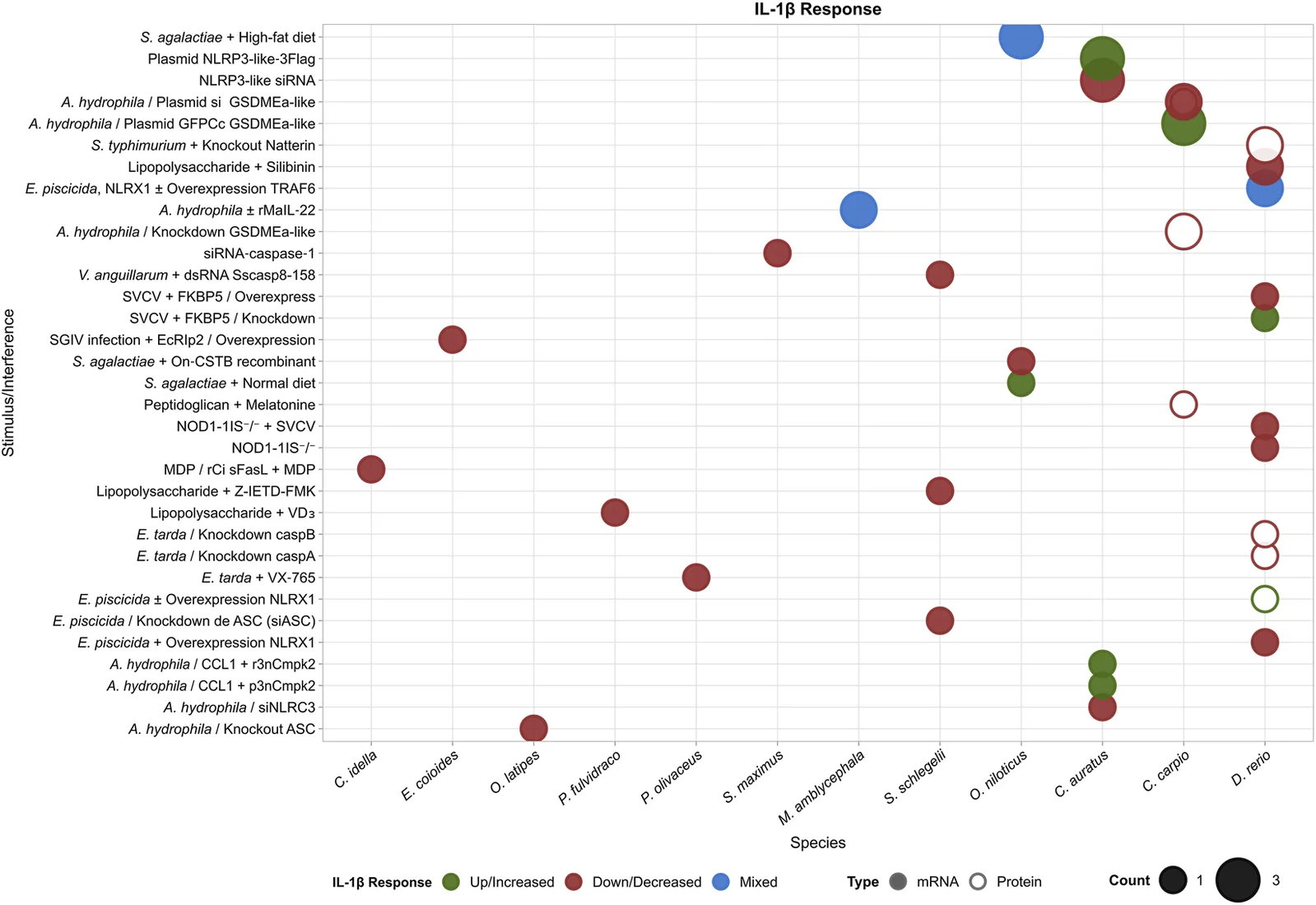
Modulation of teleost IL-1β pathways under pathogen challenge, functional interference, and dietary treatments. Comprehensive mapping of IL-1β dynamics in teleost species subjected to microbial challenges combined with functional gene knockouts, siRNA knockdowns, overexpression, chemical inhibitions, or dietary interventions (e.g., siRNA, plasmid-mediated expression of GSDME-like variants, microRNA mimics, and secondary metabolites such as silibinin or melatonin). Response direction is color-coded: green (upregulated), red (downregulated), or blue (mixed). Symbol fill indicates analysis level (solid: mRNA; hollow: protein), and bubble size represents total study counts per condition.

Figure 5 was constructed using data from Supplementary Table S5, which compiles all studies evaluating gasdermin expression (mRNA and protein) following stimulation by pathogens or their derivatives. The final layout was designed using Inkscape software (version 1.4.3). All fish illustrations featured in this figure were generated using Gemini Pro. Figure 6 maps the landscape of IL-1β regulation across teleost fishes under various environmental and physical stressors, using the curated dataset in Supplementary Table S3. Data were categorized and visualized as a multi-panel bubble chart via the *ggplot2* package in R software (version 4.4.0). Within each panel, the x-axis groups the broad categories of applied stimuli (e.g., Heavy metals, Emerging contaminants, Pesticides, Pharmacological agents, Diet and metabolism, Genetic manipulation, Immune stimulus, Hypoxia, and Physical and environmental stressors). The y-axis specifies the evaluated tissue or life stage category (Liver, Head kidney, Kidney, Intestine, Gill, Spleen, Skin, Fin, Larvae, Embryo, or Other tissue).

**FIGURE 5:**
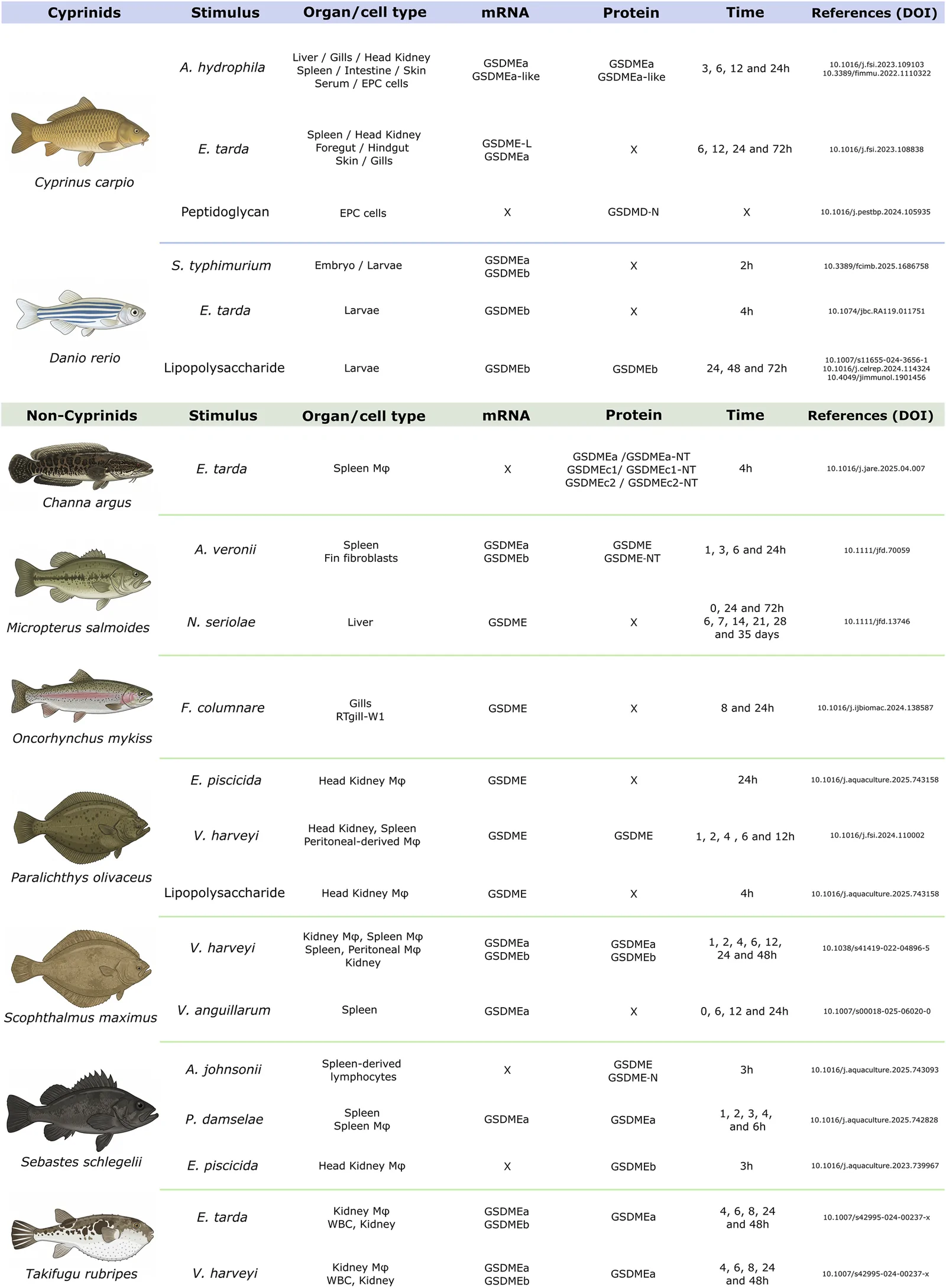
Summary of Gasdermin expression and activation dynamics in teleost fish following infectious or immunostimulatory challenges. Systematic compilation mapping experimental evidence of Gasdermin family members (mRNA and protein levels) across cyprinid and non-cyprinid models. The panel details microbial stimuli, target tissues/cell lines (including specialized macrophages, fibroblasts, and lymphocytes), molecular targets (GSDMEa, GSDMEb, GSDMD, and N-terminal cleaved fragments), sampling timelines, and literature citations. Fish schematics represent key species: *Cyprinus carpio*, *Danio rerio*, *Channa argus*, *Micropterus salmoides*, *Oncorhynchus mykiss*, *Paralichthys olivaceus*, *Scophthalmus maximus*, *Sebastes schlegelii*, and *Takifugu rubripes*. “X” indicates parameters not evaluated within the respective study designs.

**FIGURE 6:**
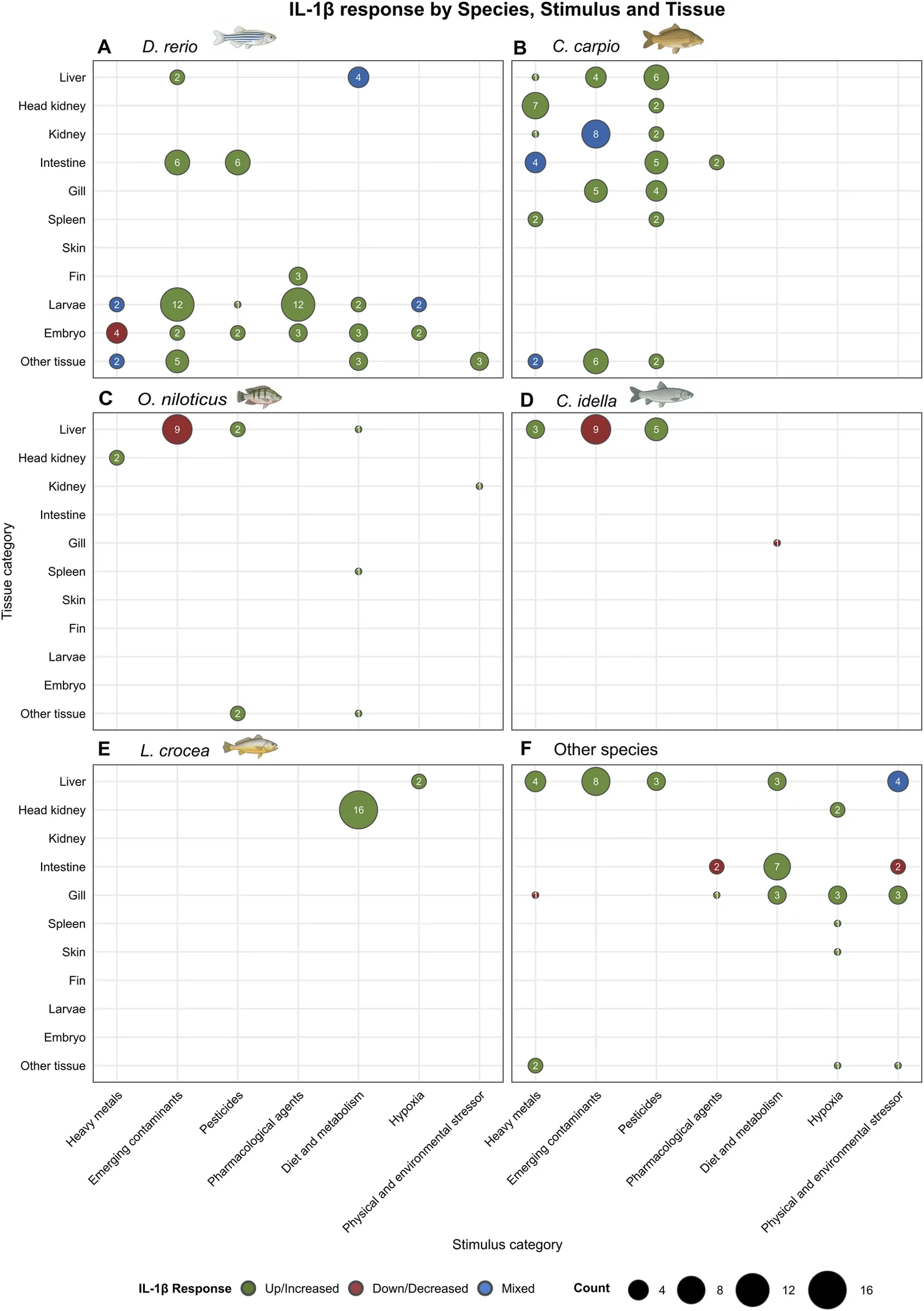
Global profile of IL-1β regulation across teleost species, tissues, and experimental stressors. Multi-panel representation for **(A)** *Danio rerio*, **(B)** *Cyprinus carpio*, **(C)** *Oreochromis niloticus*, **(D)** *Ctenopharyngodon idella*, **(E)** *Larimichthys crocea*, and **(F)** other teleost species. The x-axis categorizes experimental stressor types, while the y-axis lists evaluated tissues or life stages. Bubble colors encode IL-1β modulation: green (upregulation), red (downregulation), and blue (mixed/conflicting). Bubble size is proportional to observation count.

Figure 7 synthesizes published evidence on teleost gasdermin expression and activation under chemical or environmental challenges, using Supplementary Table S8. Data were extracted and categorized by fish species, experimental stimulus, targeted gasdermin isoform (GSDMD, GSDME, or their respective N-terminal cleaved domains), response direction, and study frequency. Species were hierarchically grouped on the y-axis according to taxonomy (Cyprinids: *Cyprinus carpio*, *Ctenopharyngodon idella*, *D. rerio*; Non-Cyprinids: *Channa punctata*, *Oryzias melastigma*), with specific chemical stimuli listed per species block. Target gasdermin genes and protein cleavage fragments were aligned along the x-axis (GSDMD, GSDMD-N, GSDME, GSDMEa, GSDMEa-N, GSDMEb, and GSDMEb-N). The multi-parameter bubble matrix was plotted using the *ggplot2* package in R software (version 4.4.0). Response orientation was color-coded (green for upregulation/increased protein cleavage; red for downregulation/decreased response), and study representation count (ranging from 1 to 3 independent observations) was mapped to point area diameter (scale_size_area). Alternating background shading was applied across species blocks to enhance visual scannability.

**FIGURE 7:**
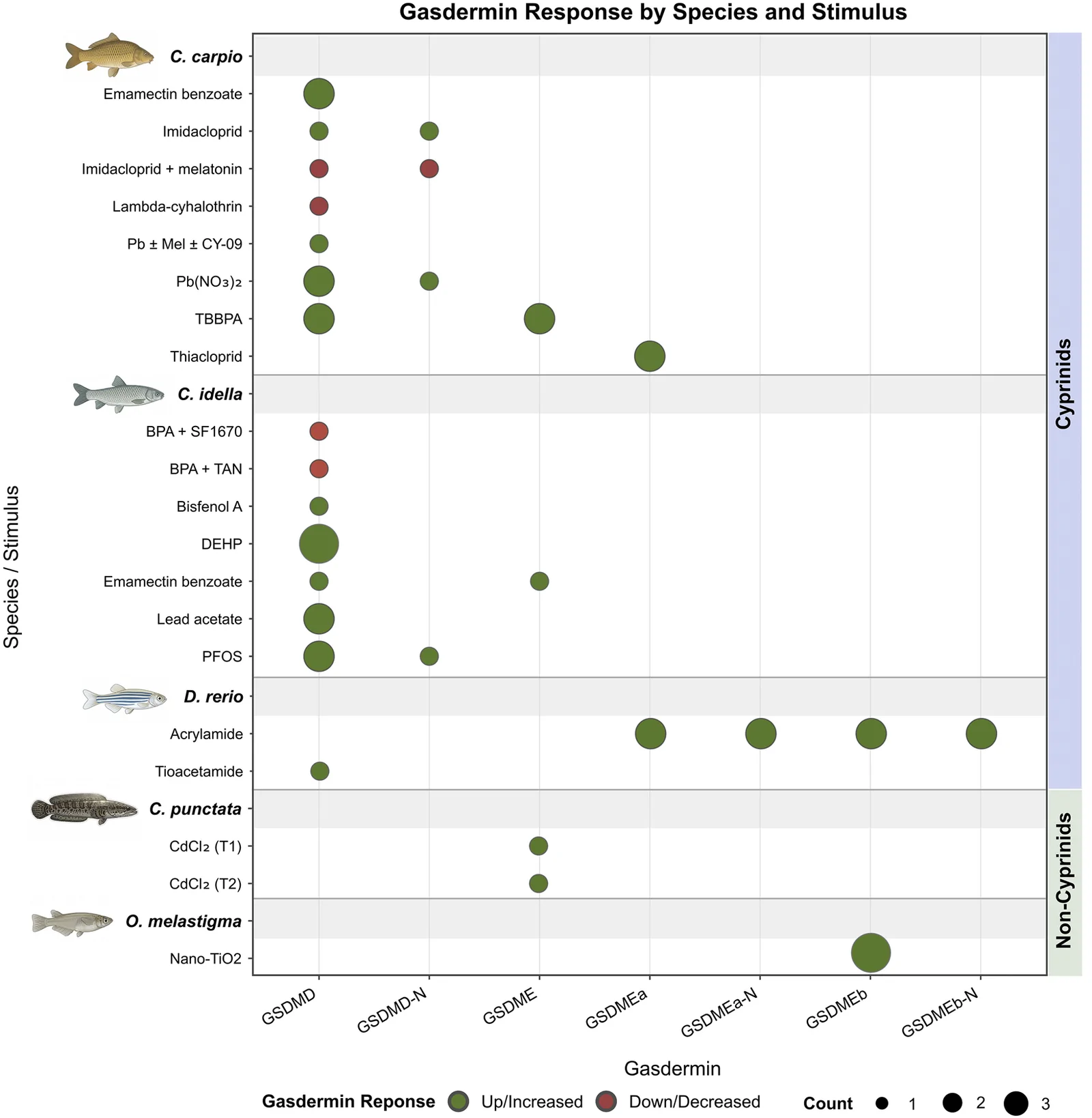
Teleost gasdermin transcription and cleavage profiles under environmental and chemical stressors. Bubble plot matrix summarizing gasdermin modulation (GSDMD, GSDMD-N, GSDME, GSDMEa, GSDMEa-N, GSDMEb, and GSDMEb-N) across five teleost species (*Cyprinus carpio*, *Ctenopharyngodon idella*, *Danio rerio*, *Channa punctata*, and *Oryzias melastigma*) categorized by taxonomic clade (Cyprinids vs. Non-Cyprinids). Rows represent specific chemical challenges; columns denote individual gasdermin isoforms and N-terminal cleavage domains. Point color indicates response direction (green: upregulation/increased cleavage; red: downregulation/decreased response). Symbol fill distinguishes detection level (solid: mRNA; hollow: protein), and bubble size reflects cumulative study count (1 to ≥3).

## 3 Results

### 3.1 Publication trends and the inflammasome paradox

The research landscape surrounding innate immunity and the inflammasome in teleosts is characterized by remarkable historical dynamism. Between 1948 and 2019, literature in this field grew slowly, accumulating 539 studies at an average of fewer than eight articles per year (data excluded from the final review). However, from 2020 to early 2026, the field experienced a growth, yielding 801 new studies in just 6 years (Figure 1; Supplementary Table S1).

Approximately 60% of all literature on fish innate immunity and the inflammasome has been published in the current decade. This massive data accumulation highlights a critical paradox: while the field is highly proficient in measuring innate immunity through soluble mediators like cytokines, exploration of inflammasome complex components, such as gasdermins, has only recently begun. For instance, out of 184 articles evaluating the activation of IL-1β expression, only 30 investigated pathogen-induced outcomes. The vast majority addressed abiotic stimuli, including drugs, pesticides, temperature, nanoparticles, dietary changes, environmental contaminants, disinfectants, oxygen levels, transport stress, and genetic interventions. This distribution demonstrates that while IL-1β is a classic inflammatory marker for host-pathogen interactions, it is now extensively utilized to gauge responses to general environmental and anthropogenic stressors.

### 3.2 Taxonomic distribution in teleost immunity research

An analysis of publication volume reveals a distinct distribution between cyprinid and non-cyprinid groups (Figure 2). At the top of the dendrogram sits a well-defined Tetraodontidae cluster, comprising six species (*Takifugu septentrionalis*, *Takifugu fasciatus*, *Takifugu sinensis*, *Takifugu rubripes*, *Takifugu obscurus*, and *Takifugu nigroviridis*). This is followed by monospecific families, including Serrasalmidae, Gadidae, Percidae, Notopteridae, and Psychrolutidae. The Bagridae family is represented by four species (*Pelteobagrus vachelli*, *Pelteobagrus ussuriensis*, *Leiocassis reissneri*, and *Tachysurus fulvidraco*).

Intermediate branches feature monospecific families such as Sparidae, Clariidae, Sinipercidae, Carangidae, Poeciliidae, Epinephelidae, and Adrianichthyidae, alongside the Moronidae family (*Lateolabrax japonicus* and *Dicentrarchus labrax*), Sciaenidae (*Nibea albiflora*, *Miichthys miiuy*, and *Larimichthys crocea*), and Channidae (*Channa punctata* and *Channa argus*). Studies also feature species from the Polynemidae, Cynoglossidae, Ictaluridae, Centrarchidae, Serranidae, and Paralichthyidae families.

Within the Cyprinidae family, 12 species were investigated for innate immune responses: *Onychostoma macrolepis*, *Megalobrama amblycephala*, *Sinocyclocheilus grahami*, *Anabarilius grahami*, *Mylopharyngodon piceus*, *Hypophthalmichthys molitrix*, *Sinocyclocheilus prenanti*, *Labeo rohita*, *Carassius auratus*, *C. idella*, *C. carpio*, and *D. rerio*. Finally, the base of the dendrogram includes species such as *Oreochromis punctatus*, *Oreochromis niloticus*, *Salmo salar*, and *Oncorhynchus mykiss*, along with members of the Sebastidae and Scophthalmidae groups.

Research focus differs noticeably between these clades. Publications on cyprinid fish predominantly investigate a broad spectrum of innate immune pathways (TLR, MyD88, TNF, IRF, IFN, NOD, ASC, NLRC, NLRP, caspases, IL, gasdermin, and CXCL). In contrast, studies on non-cyprinid fish have historically focused more narrowly on the identification of TNF and cytokines (IL). Also, we quantified in percentage the number of articles per specie as demonstrated in Supplementary Figure S1.

The immense economic value of *C. carpio*, *O. mykiss*, and *O. niloticus* in global aquaculture, alongside the importance of *D. rerio* as a model organism, drives the majority of current knowledge. Consequently, our molecular understanding of teleost innate immunity and inflammasome activation is largely extrapolated from these key species, leaving the pathways of numerous fish families unexplored.

### 3.3 Specific IL-1β response profiles to pathogens

We systematically mapped the specific IL-1β response profiles of cyprinid and non-cyprinid teleosts exposed to aquaculture-relevant pathogens and bacterial components (Figure 3).

Within cyprinids, *D. rerio* accounted for the highest volume of literature, providing a comprehensive characterization of IL-1β dynamics across multiple stimuli (Figure 3A). Exposure to lipopolysaccharide (LPS) in *D. rerio*, *L. rohita*, and *C. idella* yielded the most robust and consistently replicated upregulation clusters, predominantly at the transcriptional (mRNA) level. *Aeromonas hydrophila* infection also emerged as a potent inducer of IL-1β across all analyzed cyprinids, though *C. auratus* exhibited a mixed response. Notably, cyprinid responses were almost exclusively characterized by upregulation or mixed profiles, with no instances of strict pathogen-induced downregulation recorded.

Among non-cyprinid species (Figure 3B), LPS and *A. hydrophila* were the most widely studied stimuli, consistently driving increased IL-1β production at both mRNA and protein levels in species such as *O. mykiss*, *Paralichthys olivaceus*, and *Gadus morhua*. While pathogens like *Edwardsiella piscicida* and *Vibrio anguillarum* robustly triggered IL-1β synthesis, mixed or downregulated responses were occasionally documented. For example, specific downregulation was observed in *O. mykiss* challenged with *Ichthyophthirius multifiliis* and in *Cynoglossus semilaevis* infected with *Vibrio harveyi*. These exceptions suggest distinct temporal kinetics in host tissues or specific immune evasion mechanisms employed by the pathogens.

### 3.4 Regulatory mechanisms of the IL-1β pathway

Functional assays provided robust insights into how IL-1β production is regulated in teleosts (Figure 4). Studies employing genetic loss-of-function models targeting core inflammasome components (*nlrp3*, *gsdme*, *natterin*, *traf6*, *il22*, *caspase-1*, *caspase-2*, *caspase-8*, *nod*, *fasl*, *nlrx1*, *asc*, and *cmpk2*) consistently demonstrated a downregulation of the IL-1β response. Similarly, experimental interventions modulating circadian rhythms or metabolism—such as melatonin administration, high-fat diets, or *cmpk2* targeting—resulted in the attenuation of IL-1β at both the mRNA and protein levels.

### 3.5 Gasdermin expression and cleavage dynamics

Gasdermin (GSDM) proteins act as the primary executioners of pyroptosis (an inflammatory form of lytic programmed cell death) and coordinate innate immunity by facilitating IL-1β release and K+ efflux (Evavold et al., 2018; Rühl and Broz, 2022). Our analysis of GSDM expression (Figure 5) revealed the following. In Cyprinids, research focused primarily on GSDMEa and GSDMEb, showing sustained expression ranging from 3 to 72 h post-infection (hpi) in *C. carpio* and *D. rerio*. In Non-cyprinids, studies demonstrated expression between 1 and 4 hpi in *C. argus* and *Sebastes schlegelii*, including the recruitment of GSDMEc isoforms.

It must be noted that numerous studies (marked with an “X”) verified only transcriptional expression (mRNA) without corroborating proteomic analysis of the cleaved, active protein. These methodological gaps likely reflect specific study scopes rather than a biological absence of functional GSDMs. Gasdermin cleavage provides an additional functional readout by demonstrating proteolytic activation of the precursor into its pore-forming effector fragment, thereby distinguishing increased expression from downstream functional activation (Cao and Kagan, 2022; Zhao L. et al., 2023).

### 3.6 Tissue-specific toxicological responses

A multi-panel plot revealed distinct species- and tissue-specific patterns of IL-1β regulation (Figure 6). A substantial prevalence of green bubbles indicated that upregulation is the primary global response across most tissues and stimulus categories. However, specific trends mirror the physiological utility of different model organisms. *D. rerio* (Figure 6A) serves as the primary model for developmental toxicity; In *C. carpio* (Figure 6B), the kidney and head kidney function as critical immune-regulatory sites, particularly sensitive to heavy metals and pesticides, and in *O. niloticus* (Figure 6C), research is heavily biased toward nutritional immunology, featuring a massive cluster of upregulated responses (16 counts) in the head kidney triggered by dietary and metabolic stimuli.

Prominent exceptions to this global upregulation trend occurred in *L. crocea* (Figure 6E) and *C. idella* (Figure 6D). In both species, the liver stands out as a major toxicological target, marked by distinct IL-1β downregulation (9 and 12 counts, respectively) driven largely by emerging contaminants. Conversely, in a diverse range of less-studied teleosts (Figure 6F), the liver remains the premier toxicological target but consistently exhibits robust IL-1β upregulation in response to heavy metals, contaminants, and pesticides.

### 3.7 Gasdermin responses under chemical stress

Systematic mapping of gasdermin modulation under chemical stress revealed distinct isoform- and species-specific patterns (Figure 7). Overall, GSDMD and GSDME were the primary targets evaluated, with transcriptional upregulation acting as the predominant outcome following xenobiotic exposure.

In the Cyprinids, *C. carpio* exhibited widespread upregulation of GSDMD when challenged with pesticides and heavy metals, including emamectin benzoate, imidacloprid, lead nitrate (Pb(NO_3)_2), and tetrabromobisphenol A (TBBPA). However, co-exposure to lambda-cyhalothrin or imidacloprid combined with melatonin resulted in significant downregulation of GSDMD and its active fragment, GSDMD-N. In *C. idella*, persistent organic pollutants (PFOS, lead acetate) and plasticizers (DEHP, Bisphenol A) consistently triggered GSDMD upregulation. Co-treatments with BPA and inhibitors (SF1670 or TAN) successfully suppressed GSDMD. In *D. rerio*, acrylamide exposure induced comprehensive activation across duplicated paralogs, causing robust upregulation of GSDMEa, GSDMEa-N, GSDMEb, and GSDMEb-N, whereas thioacetamide selectively upregulated GSDMD.

In the Non-cyprinids, *C. punctatus* exhibited targeted upregulation of GSDME under cadmium chloride (CdCl_2) exposure at multiple time points (T1 and T2). Similarly, *O. melastigma* demonstrated strong induction of GSDMEb following exposure to nano-TiO_2.

Crucially, across all evaluated taxa, the activation and accumulation of N-terminal functional domains (GSDMD-N, GSDMEa-N, and GSDMEb-N) perfectly mirrored the directional regulation of their respective full-length precursor genes.

## 4 Discussion

In this review, we evaluate the literature published between 2020 and 2026 to characterize the IL-1β-dependent innate immune response across cyprinid and non-cyprinid teleosts. The majority of this research focuses on cyprinids, heavily driven by the use of *D. rerio* as a premier model organism in biomedical sciences (Choi et al., 2021; Patton et al., 2021) and toxicology (Lopes-Ferreira et al., 2021; Dasgupta et al., 2022), facilitated by its fully sequenced genome (Howe et al., 2013). Beyond zebrafish, species of immense global aquaculture value—such as *C. carpio*, *C. idella*, *O. niloticus*, and *O. mykiss*—comprise a significant portion of the literature, driven by the need to understand immune responses against pathogens in intensive farming (Liu R. et al., 2022; 2025b; Zhao et al., 2022; 2023c). This dual prominence provides a vast and robust dataset on how teleosts respond to various challenges.

Notably, 96.7% of the reviewed literature (178 out of 184 studies) reported a significant modulation of IL-1β under diverse stimuli, highlighting a highly plastic activation mechanism. In infectious models, studies across species consistently demonstrate the evolutionary conservation of the “canonical” NLRP3 inflammasome pathway, which triggers gasdermin-dependent pore formation for antibacterial defense (Huang et al., 2025; Donoso et al., 2026; Li et al., 2026; Mathiessen et al., 2026; Xu et al., 2026).

However, our systematic analysis reveals a critical paradigm shift: the vast majority of recent manuscripts target abiotic stressors typical of environmental toxicology rather than infectious agents (Zhang et al., 2022; Zhang Y. et al., 2025; Guo et al., 2023; Zhou et al., 2023; Miao et al., 2024; Shi et al., 2024; Diao et al., 2025; Lou et al., 2025; Saxena et al., 2025; Zheng et al., 2025). While pathogens activate IL-1β via PAMPs, abiotic factors primarily operate by inducing severe oxidative stress and overproducing Reactive Oxygen Species (ROS). Exposure to sublethal concentrations of heavy metals—such as cadmium (Zhang et al., 2020; Li Y. et al., 2022; Saxena et al., 2025), lead (Jing et al., 2020), and copper (Zhou et al., 2023; Zhang Y. et al., 2025)—as well as commercial pesticides (Zhang et al., 2022; Gong et al., 2023; Liu et al., 2023; Wang W. et al., 2024; Han et al., 2025) causes mitochondrial dysfunction and cellular injury. This damage releases Damage-Associated Molecular Patterns (DAMPs) that trigger NLRP3 activation.

Consequently, the IL-1β axis acts as a central hub responding to xenobiotics, extreme environmental conditions (hypoxia, temperature, and salinity fluctuations), and even dietary shifts (high-fat diets). These stimuli modulate key inflammatory components—including ASC, caspase-1, NLRP3, NLRC3, and NF-κB—as demonstrated in studies using pesticides like chlorpyrifos and avermectin, or substances like nigericin (Morimoto et al., 2021; Zhang J. et al., 2021; Zhang J. et al., 2025; Zou et al., 2021; Costa et al., 2022; Du et al., 2022; Li J. et al., 2022; Liu E. et al., 2022; 2023; 2024; Moniruzzaman et al., 2022; Bi et al., 2023; Wang Z. et al., 2024; 2025b; Wan et al., 2024; Zhang et al., 2024; Jia et al., 2025; Miao et al., 2025; Tao et al., 2025; Wu et al., 2025). Ultimately, IL-1β transcription serves as an ultra-sensitive biomarker for tissue inflammation, bridging environmental pollution and immune system disruption.

When analyzing infectious models, distinct pathogens trigger innate immunity through the Toll-like receptor (TLR) and MyD88 axis (Xin et al., 2020), alongside NOD1 (recognizing iE-DAP, primarily in Gram-negative bacteria) and NOD2 (recognizing MDP, a sensor for both Gram-positive and Gram-negative bacteria) (Li H. et al., 2022; 2022c; 2022d; Li X. et al., 2022; Mahapatra et al., 2023; Chen et al., 2025; Pakhira et al., 2025; Wang J. et al., 2025; 2025c).

Species-specific regulatory networks further highlight this complexity. In *Clarias gariepinus* head kidney macrophages exposed to *A. hydrophila*, defense involves TLR22 (Kumar et al., 2021), Caspase-3 (Sharma et al., 2023), and the TNF-α/Caspase-b/IL-1β axis (Sun J. et al., 2022; Yang et al., 2022; Gong et al., 2023). In *O. niloticus*, the antibacterial response relies on IFN-γ and IL-18-dependent upregulation (Bi et al., 2023; Dinakaran et al., 2024). Interestingly, NLRC3 has been shown to play a crucial role in the antibacterial defense of *C. semilaevis*, *Lateolabrax maculatus*, and *Scophthalmus maximus* (Qi et al., 2021; Zhang L. et al., 2021; Sun R. et al., 2022; Pan et al., 2023; Yuan et al., 2023), starkly contrasting with its typical inhibitory role in mammals (Eren et al., 2017; Xu et al., 2023).

Our analyses confirm that specific NLRs function as critical sensors for the IL-1β response: NLRP3 is vital in *Micropterus salmoides* and *P. olivaceus* (Wang X. et al., 2021; Guo et al., 2025; Li et al., 2026), while NLRC4 is key for *Epinephelus coioides* (Zhao L. et al., 2023).

The absolute structural requirement of the NLR/ASC/caspase platform has been definitively proven. In *Oryzias latipes*, ASC knockout completely abrogates IL-1β signaling in the kidney after 24 h (Morimoto et al., 2020) by dismantling the required caspase-activation scaffold. Similarly, siRNA-mediated silencing of caspase-1 in intestinal epithelial cells of *S. maximus* significantly reduced IL-1β mRNA levels within 24 h (Cui et al., 2023). Furthermore, *in vitro* experiments utilizing a recombinant expression plasmid for cytidine monophosphate kinase 2 (Cmpk2) from a triploid *C. auratus* clone (r3nCmpk2) induced an immediate increase in IL-1β within 1 h (Feng et al., 2021), consistent with mitochondrial DNA oxidation acting as a rapid intracellular danger signal.

Because pro-IL-1β lacks the signal peptide for conventional ER-to-Golgi secretion, it relies exclusively on gasdermin pores to exit the cell (Shi et al., 2017; Xia et al., 2020; Wang H. et al., 2023; Zhao et al., 2023b). Due to the teleost Whole Genome Duplication (WGD) event, the *gsdm* gene expanded into multiple paralogs, primarily GSDMEa, GSDMEb, and GSDMEc.

Current literature establishes GSDME as the primary mediator of inflammatory cell death in teleosts (Chen H. et al., 2021). The robust identification of active N-terminal (NT) fragments for the “a, b, and c” isoforms following bacterial challenges (e.g., *V. harveyi*, *Edwardsiella tarda*) confirms their role as the molecular “switch” for pore formation and pro-inflammatory lysis (Xu et al., 2024; 2026). However, an evolutionary division of labor appears to exist: while GSDMEa and GSDMEb execute pyroptotic cell death, GSDMEc variants frequently produce non-pyroptotic cleavage products or remain inactive under identical stimulation (Xu et al., 2026). This diversification likely reflects a strategy to finely modulate the threshold and intensity of pyroptosis (Xu et al., 2022; Xu et al., 2024; Xu et al., 2026; Wang H. et al., 2023; Zhao et al., 2023c; 2023b; Miao et al., 2024; Ding et al., 2025; Guo et al., 2025; Gusso et al., 2025; Jin et al., 2026).

Although 13 species groups have been analyzed, only 9 show definitive evidence of gasdermin activation at the protein level. Species like *O. mykiss* and certain variants of *C. carpio* exhibit robust transcriptional induction but lack confirmed cleavage data, marking critical areas for future functional research (Li et al., 2020; 2026; Wang et al., 2020; Wang H. et al., 2023; Wang X. et al., 2023; Wang Z. et al., 2024; 2026; Xu et al., 2022; 2024; 2026; Zhao et al., 2023c; 2023b; Hao et al., 2024; Miao et al., 2024; Ou et al., 2024; Ding et al., 2025; Guo et al., 2025; Gusso et al., 2025; Nie et al., 2025; Song et al., 2025; Jin et al., 2026). Intriguingly, recent findings in *C. carpio* revealed a GSDMEa-like variant that lacks typical cytotoxic pore-forming activity *in vitro*, yet its silencing drastically impairs IL-1β secretion and bacterial clearance (Fang et al., 2022; Kong et al., 2023; Hao et al., 2024). This functional specialization suggests that gasdermins act as terminal executioners of a finely orchestrated response that can transcend simple cell lysis.

The systematic mapping of gasdermin (GSDM) expression provides powerful insights into how environmental pollutants trigger inflammatory cell death. GSDMD and GSDME are the primary functional targets evaluated, with transcriptional upregulation being the predominant outcome of chemical stress.

Among cyprinids, *C. carpio* exhibits widespread GSDMD upregulation when exposed to pesticides and heavy metals, including emamectin benzoate, imidacloprid, lead nitrate, and tetrabromobisphenol A (TBBPA). Crucially, co-exposure to protective compounds such as melatonin, or co-treatments involving lambda-cyhalothrin, successfully attenuated this toxicity, significantly downregulating both GSDMD and its pore-forming fragment, GSDMD-N. A parallel protective effect was observed in *C. idella*, where GSDMD upregulation driven by plasticizers (DEHP, Bisphenol A) and persistent pollutants (PFOS, lead acetate) was suppressed using specific pathway inhibitors (SF1670 or TAN). In *D. rerio*, acrylamide exposure induced comprehensive activation across duplicated paralogs, triggering GSDMEa, GSDMEb, and their active fragments (GSDMEa-N and GSDMEb-N), while thioacetamide selectively upregulated GSDMD.

Non-cyprinids exhibited distinct, isoform-specific responses. *Channa punctata* displayed time-dependent GSDME upregulation under cadmium chloride exposure, whereas *O. melastigma* showed robust GSDMEb induction following titanium dioxide nanoparticle treatment. Across all taxa, the accumulation of active N-terminal domains perfectly mirrored the regulation of their precursor genes. By characterizing gasdermin cleavage patterns as sensitive molecular indicators of responses to xenobiotic exposure, this work supports their potential utility as biomarkers for aquatic ecotoxicological risk assessment, while recognizing that further validation is required to establish their reliability and predictive value.

Bibliometric analysis showed that approximately 60% of the historical literature on teleost innate immunity and inflammasome mechanisms was published between 2020 and 2026, highlighting the substantial expansion of research in this field. This growing body of evidence encompasses a diverse range of teleost species, including established laboratory models and economically important aquaculture species, providing an increasingly broad comparative basis for investigating the conservation and diversification of inflammasome-associated pathways. The increasing number of publications reflects growing scientific interest in inflammasome-related responses in teleosts but does not, by itself, establish the efficacy or predictive value of the IL-1β/Gasdermin axis as a toxicity biomarker.

Functionally, the IL-1β axis has emerged as a highly integrated and extraordinarily plastic biosensor. Canonically, IL-1β production is triggered by key aquatic pathogens via conserved TLR/MyD88 and NOD1/NOD2 signaling pathways. However, this axis is also acutely sensitive to a broad spectrum of abiotic, environmental, and anthropogenic stressors, including pesticides and heavy metals. In zebrafish embryo assays, this environmental sensitivity may provide a promising mechanistic window. Characterizing the functional conservation of the canonical NLRP3/ASC/Caspase platform could help determine whether these pathways may serve as molecular indicators of cellular stress and provide early mechanistic information preceding the manifestation of macroscopic developmental abnormalities.

Finally, teleost Whole Genome Duplication (WGD) has driven the evolutionary diversification of gasdermin (GSDM) paralogs, adding a unique layer of regulatory complexity. While GSDMEa and GSDMEb serve as the primary executioners of pyroptosis and the terminal coordinators of IL-1β release, GSDMEc variants exhibit non-canonical functions that finely tune the inflammatory threshold. Despite these advances, a critical gap remains: the field currently relies too heavily on transcriptional (mRNA) data, underscoring an urgent need for the proteomic verification of gasdermin and caspase cleavage. Bridging this proteomic gap in *D. rerio* embryos may represent an important step toward improving the mechanistic resolution of developmental toxicity screening. Characterizing GSDMEa/b cleavage products as potential functional biomarkers could complement descriptive morphological assessments with higher-resolution, mechanism-based endpoints and may contribute to the development of more predictive approaches in environmental toxicology. However, their predictive performance and relevance to subsequent developmental outcomes require further experimental validation. Future studies should complement transcript-level analyses with direct assessment of Gasdermin cleavage at the protein level, using appropriate biochemical or proteomic approaches. Such validation would help determine whether transcriptional changes are accompanied by functional activation of these pathways and clarify their potential relevance to toxicity assessment. However, GSDM cleavage profiling remains a relatively costly and time-consuming approach, which may limit its practical application in high-throughput toxicity assessment.

## Data Availability

The original contributions presented in the study are included in the article/Supplementary Material, further inquiries can be directed to the corresponding author.

## Supplementary material

SUPPLEMENTARY FIGURE S1 Distribution of unique studies across fish species included in the systematic review. Values next to the bars indicate the absolute number of unique studies followed by the corresponding percentage relative to the total number of included articles. Species with only one study were omitted from this figure for clarity. The data used to generate this figure were obtained from Spreadsheet 2 of Supplementary Table 4.
